# Paleo-climatic control on recharge and fresh-salt groundwater distribution in the Red River delta plain, Vietnam

**DOI:** 10.1038/s41598-024-71899-x

**Published:** 2024-09-11

**Authors:** Flemming Larsen, Hoan Van Hoang, Long Vu Tran, Nhan Quy Pham

**Affiliations:** 1 Geological Survey of Denmark and Greenland, Øster Voldgade 10, 1350 Copenhagen, Denmark; 2 National Center for Water Resources Planning and Investigation, Long Bien District, Sai Dong Ward, 1000 Hanoi, Vietnam; 3 Department of Hydrogeology, Hanoi University of Mining and Geology, Hanoi, Vietnam; 4 Hanoi University of Natural Resources and Environment, Hanoi, Vietnam

**Keywords:** Environmental sciences, Hydrology

## Abstract

Paleo-climatic induced sedimentation controls present-day recharge and the fresh-salt groundwater distribution in Quaternary delta systems. During sea-level highstands, marine clays with saline pore water were deposited and are interbedded with aquifers of coarse-grained sandy fluvial and shallow marine deposits, laid down during lowstands. The low-permeable marine layers may inhibit recent recharge to deeper aquifers, and thereby limit sustainable use of these freshwater resources. This phenomenon has been investigated in the Red River delta plain, using geophysical borehole logging, transient electromagnetic soundings, groundwater chemistry, stable isotope analysis and ^3^H and ^14^C dating of groundwater. Results reveal that marine saline pore water is still present in the Holocene marine clays, implying that fresh water has not entered the clays since their deposition. Therefore, recharge within the delta plain is not occurring and the deeper aquifers are hydraulically disconnected from the upper sandy layers. Today, recharge only occurs from the hinterland. Recharge during the last glacial period has flushed saline pore water from Pleistocene marine clays, but these clays were again affected by saline water during the Holocene transgression. The use of the groundwater resources in the delta plain must be adjusted to the present recharge to be sustainable.

## Introduction

Nearly a quarter of the world’s population lives within 100 km of a coast-line^1^, and fresh water is essential for sustaining these populations and near-coastal ecosystems. However, coastal water resources are threatened by saltwater intrusion due to over-pumping and climate change^2^, and a strategy for management of these resources is needed to avoid exhaustion^3^. Several studies published during the past decade have shown that the hydrological conditions in Holocene coastal and deltaic aquifer systems have not reached hydraulic equilibrium with respect to salinity distribution with the present-day sea-level stand^4–11^. Therefore, the occurrence of salty groundwater in these delta systems can only be understood by considering how and when deposits were laid down using paleo-hydrological modelling^3,8^. Recharge of fresh water in many delta systems occurred during the last glaciation, when large hydraulic gradients were created inland due to glacially lowered sea-level. For a more detailed elaboration of this, see *Morrissey *et al.^4^. If recharge to the Quaternary coastal aquifers occurred under larger hydraulic gradients in the past, we must pose the question—what is the present recharge to these aquifers, given the present lower hydraulic gradients, and is the water abstraction sustainable^3^?

We have studied this issue in the Nam Dinh province in the Red River delta plain in Vietnam, where groundwater has been abstracted since the beginning of the 1990s, and questions on the sustainability of this groundwater use have been raised.

To answer this question, we must elucidate the hydraulic properties of the marine clay deposits. Quaternary clastic coastal and deltaic depositional systems often contain thick sequences of Holocene marine mud, deposited during transgressions at eustatic high stands^12^. The permeability of the marine mud, which are aquitards in the delta systems, is a critical parameter controlling groundwater flow and recharge^13,14^. An aquitard refers to a consolidated or unconsolidated clay or mud formation with low permeability, that under ordinary hydraulic gradients transmits a quantity of water significant for regional groundwater flow^15^. Laboratory and field data of the hydraulic properties of aquitards have shown lower hydraulic conductivity, or permeability, than previously anticipated^11,14^. Reported hydraulic conductivities (K) in the literature are between 10^–16^ m/s and 10^–8^ m/s and are related to porosities such that a decrease in porosity of 13%, due to compaction under burial, results in an order of magnitude decrease of K^16^. A review of the literature of homogeneity of K values shows no scale effect from the laboratory to the field , but variations in K are observed on a regional scale^13^.

Studies have been conducted focused on the effect of aquitards on saltwater intrusions in aquifers^14,17^, and on the control exerted by low-permeable formations on the depth of the interface between the saltwater to freshwater interface, its position, and distance from the shoreline^18^. Aquitards also influence the dynamics of submarine groundwater discharge^19^. More recent studies address the impact of a climatically induced sea-level rise on sea-water intrusion in aquifers^20,21^, and on the effect of sea-level rise in recharge-limited coastal groundwater systems^22^.

We have previously reported results of regional modelling of saltwater flushing of saline porewater from Holocene marine mud in the Red River delta plain^8^, and a study of this process has been conducted in a single borehole in the North China Plain, influenced by a Holocene marine transgression^11^. With data from a multidisciplinary study, we here show in detail the impact of aquitards on recharge in coastal regions, and on sediment hydraulic properties controlling the widely reported hydraulic disequilibrium in Quaternary delta systems. The results yield a general contribution to the understanding of groundwater recharge mechanisms in coastal regions and the management of these water resources.

## Geology and hydrogeology

**Fig. 1:**
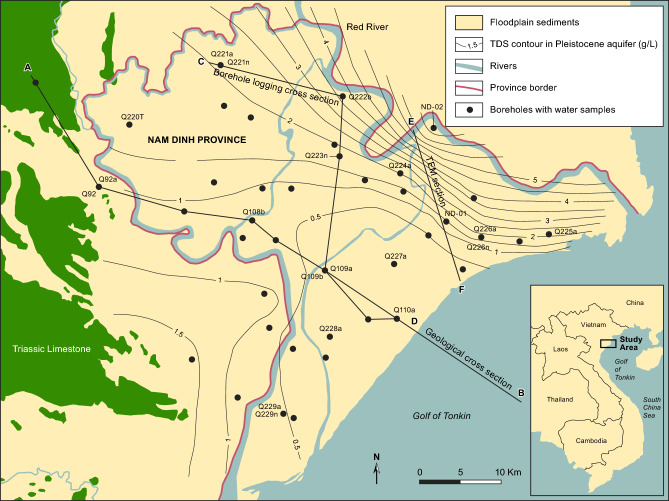
The Nam Dinh province of the RRDP. Contours show concentration of total dissolved solids (TDS) in groundwater. Line AB shows the location of the geological cross-section in Fig. 2; CD shows the location of the borehole logging section in Fig. 5; EF shows the location of transient electromagnetic measurements in Supplementary Information Fig. S1. Boreholes where groundwater was sampled are also indicated. Map background produced in MapInfo Pro 15.0 using data from National Center for Water Resources Planning and Investigation. Contours produced by Surfer v. 24.

The Nam Dinh province is a part of the Red River delta plain (RRDP), located south of the Red River outlet to the Gulf of Tonkin (Fig. 1). The Quaternary sediments of the RRDP, where fully developed and preserved, comprise five units of coarse-grained fluvial deposits and fine-grained marine sediments composed of clay, silt, and fine sand with organic material^23^. This stratigraphy reflects the eustatic sea-level changes through the Quaternary period, with a low sea-level 130 m below present during the Pleistocene and high sea-level during the Holocene^12^. The sea-level reached a high of 3 m above present sea level in the period from 6000 to 4000 years BP^24^.

**Fig. 2:**
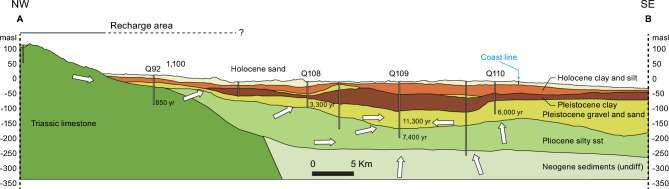
The near-surface geology and hydrogeology of the Nam Dinh province and ^14^C groundwater ages. Note that ^14^C ages are not sediment ages, but corrected ages of the groundwater; sediment ages are mentioned in the text. For location of the cross-section, see Fig. 1. The write arrows indicate the regional groundwater flow, based on measured hydraulic heads given in Fig. 3 and ages of the groundwater.

A representative transect, approximately perpendicular to the coastline, is depicted in Fig. 2. Limestones crop out towards the west and northwest, and Neogene sand, silt and claystone underlie Quaternary deposits in the central part of the study area. Pleistocene coarse-grained alluvial and fluvial deposits occur at depths from ~ 70 m to ~ 180 m below the surface (mbs), and Pleistocene marine deposits are also present. Holocene marine clays occur at depths from ~ 10 to ~ 100 mbs and ^14^C dating of peat layers in the sediments indicates a deposition age of 10,000 years (10 kyr), with depositional ages decreasing to 1 kyr towards the present coastline^24,25^. The uppermost ~ 10 m of the subsurface geology consists of Holocene delta plain and delta front deposits, composed of organic-rich deposits of sand, silt and clay (Fig. 2).

The hydrogeological setting of the Nam Dinh area is composed of an upper unconfined sandy to silty aquifer in the Holocene delta front sediments, and a lower confined aquifer in coarse- grained fluvial deposits of Pleistocene age. The two aquifers are separated by aquitards of marine Holocene and Pleistocene muds (Fig. 2). The upper unconfined sand is in hydraulic contact with a dense network of drainage canals and shallow river tributaries in the delta. The Holocene and Pleistocene marine aquitard muds are non-indurated. When fractured, the underlying limestones and the Neogene silty sandstones are aquifers in hydraulic contact with the Pleistocene aquifer.

The RRDP is in the sub-tropical monsoon zone, with a rainy season from May/June to October /November^26^. During high river stages in the monsoon, river water recharges the shallow aquifers, whereas during low fluvial stands, the aquifers discharge into the Red River^27^. During storms and low-river stages in the Red River, salty surface water is located as far inland as 35 km from the coast^28,29^.

**Fig. 3:**
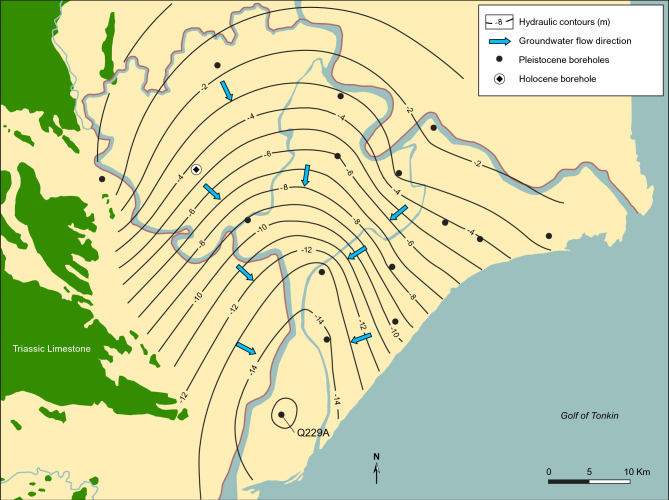
Hydraulic head in the Pleistocene aquifer. Contours show the distribution of the hydraulic head in the Pleistocene aquifer in 2023**.** Map background produced in MapInfo Pro 15.0 using data from National Center for Water Resources Planning and Investigation. Contours produced by Surfer v. 24.

**Fig. 4:**
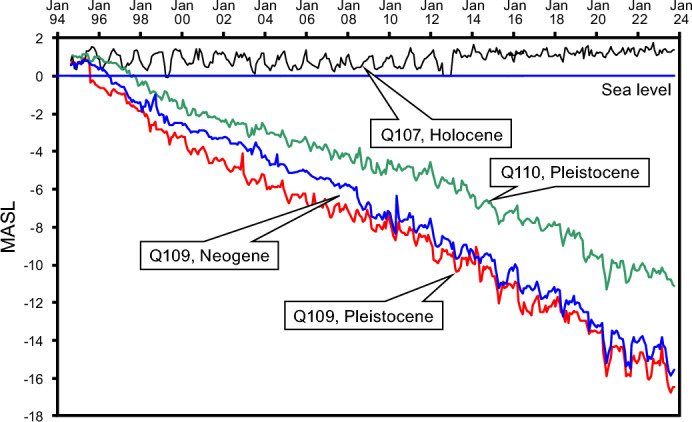
Drawdowns in the Pleistocene and Holocene aquifers. The development in the hydraulic heads in the Neogene aquifer (Q109), and Pleistocene (Q109 and Q110) and Holocene Q107 for the period from 1995 to 2023. For location of boreholes, see Fig. 1.

**Fig. 5:**
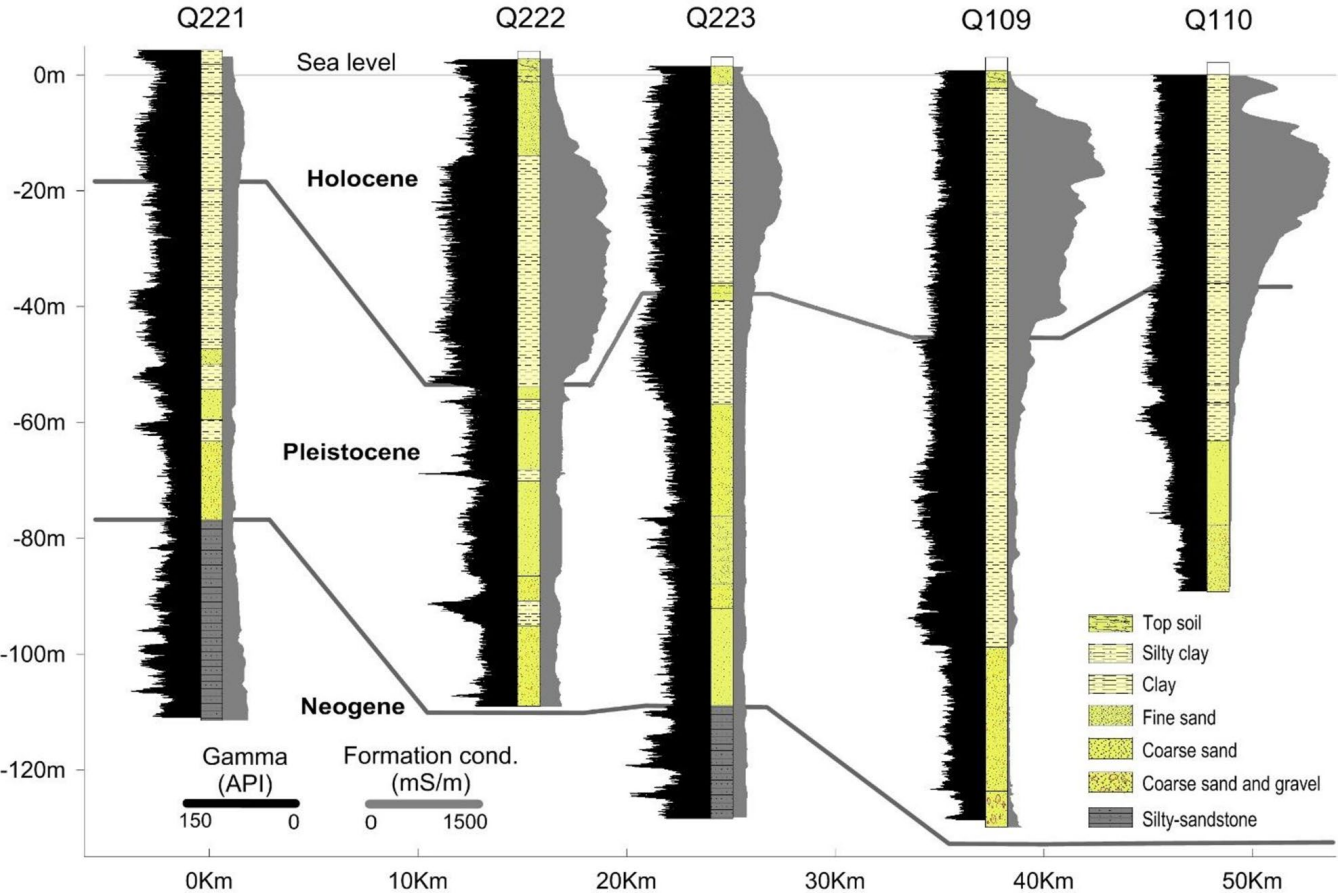
Geophysical logs in five boreholes in a line almost perpendicular to the present coastline. For the locations of borehole see Fig. 1.

Groundwater pumping from the deep, Pleistocene aquifer in the Nam Dinh province since 1996 has created a large cone of depression with the largest drawn down of 16 m around borehole Q229A (Fig. 3). Prior to the initiation of groundwater abstraction, the hydraulic heads in the deep Pleistocene and Neogene aquifers (Fig. 4, boreholes Q109 and Q110) were ~  + 1 m above sea level (masl), and the aquifer was thus artesian. In the upper Holocene aquifer, groundwater elevations were between 0 and 1 masl, with a seasonal variation controlled by the river stage (Fig. 4, borehole Q107). For much of the year, the natural hydraulic gradient across the marine mud was thus close to zero, but during dry seasons and low fluvial stages, the upward gradient was close to 1/100, as the Pleistocene and Holocene aquitard muds together are about 100 m thick (Fig. 5, borehole Q109). Groundwater pumping, initiated in 1996 from the deep, Pleistocene aquifer, led to a lowering of the groundwater heads in the aquifer down to 16 m below sea level in year 2023, and groundwater flow conditions become entirely controlled by the water abstraction (Fig. 3). In the central part of the study area, this reversed the hydraulic gradient, and a maximum downward gradient of 16/100 gradually developed.

Groundwater total dissolved solids (TDS) in the deep aquifer may exceed 5000 mg/L with the highest concentrations close to the Red River and the lowest concentrations in the central parts of the province, where groundwater is abstracted (Fig. 1). Low groundwater TDS values (1000 to 1500 mg/L) are encountered upstream near the limestone areas towards the west (Supplementary Information Table S1). In the shallow aquifer, groundwater chloride concentrations vary from 3 to 11,200 mg/L (Supplementary Information Table S2), and the stable isotopic composition of the water varies between δ^18^O -7.2 and -3.0 ‰ and δD –48.3 to –21.6 ‰ indicating that mixing of fresh groundwater and intruded oceanic water in the river distributaries controls the shallow water geochemistry (Supplementary Information Table S2). The stable water isotope of the groundwater in the Pleistocene aquifer varies within a relatively narrow δ^18^O range between -8.6 and -5.9 ‰ and δD from –60.5 to –38.5 ‰ (Supplementary Information Table S1). The difference in the isotopic compositions of the water in the two aquifers strongly suggests a different origin for the groundwaters. Furthermore, the variation in the isotopic compositions of water in the Pleistocene aquifer parallels that of the local meteoric trend, indicating that limited evaporation of the water occurred during infiltration, whereas deviation of the composition from the meteoric line of the shallow groundwater suggests that evaporation of the surface water occurred prior to recharge^30^.

Groundwater ages from ^14^C dating are between 850 and 1100 years in the recharge area and ages increase along the flow path through the Pleistocene and Neogene deposits with ages up to 11,300 years at the abstraction point (Fig. 2). Particle groundwater flow velocity from the recharge area and into the Pleistocene aquifer of 10 m/yr fits well with an increase in ages of ~ 2000 yr along the 20 km flow distance from the limestone recharge area to borehole Q108 (Fig. 2). The calculated ^14^C ages show older water in the Neogene deposits with ages of 7400 yr in boreholes Q109 and even older in the pumped section of the borehole which must be due to mixing of different groundwaters, where the older component represents deep water from the Neogene aquifer.

## Hydraulic and water chemistry composition of the marine clays

Laboratory measurements of hydraulic conductivities of the Holocene marine muds are on the order of 10^–10^ m/s and porosities of 0.3 to 0.4^8^. Using these values, and an upward hydraulic gradient of 1/100, yields an upward natural groundwater flow velocity in the mm-scale per year. Such an advective velocity is slower than transport by molecular diffusion which is typically ~ 25 cm/yr at aquifer conditions^31^, implying that transport of solutes and porewater in the Holocene marine aquitards is controlled by diffusion during pristine conditions.

Porewater compositions of the Pleistocene and Holocene muds were investigated using geophysical borehole logging measurements of formation natural gamma radiation and electrical conductivity. Representative logs from five boreholes are depicted in a transect from the sea and inland in Fig. 5. For the location of these boreholes, see Fig. 1. The aquitard mud sediments, with a high formation gamma response, are distinguished from quartz-rich aquifer sandy layers, that show a low gamma response. Salinity variations in the porewater are interpreted from measured formation conductivities in intervals where gamma radiation levels are near constant. In the most inland borehole (Q221), the Holocene marine mud is thin, and fresh or brackish groundwater is observed in both aquifers and aquitards, as indicated by formation electrical conductivities below 100 mS/m. When Holocene marine muds are thicker and overlie sandy Pleistocene deposits (as in borehole Q222) the electrical conductivity log pattern in the Holocene mud is almost symmetrical, with decreasing formation conductivities towards overlying and underlying sand layers. Such a conductivity pattern, and hence pore-water salinity distribution, strongly indicates diffusive transport of marine pore-water out of the muds. Where the Holocene marine mud is relatively thick closer to the present coastline (as in the boreholes Q109 and Q110), the measured electrical formation conductivities are typically between 500 and 1000 mS/m, with a measured peak value of the formation electrical conductivity of 1500 mS/m in borehole Q109, corresponding to groundwater chloride concentrations of 16,000 mg/L, or about 85% that of oceanic water. The almost constant and high natural gamma radiation in this formation reflects a homogeneous mud formation, and hence the very high electrical formation conductivities must be due to the occurrence of highly saline pore-water, and not a change in lithology. The asymmetrical formation electrical conductivity log pattern, with low formation electrical conductivity in the Pleistocene clay, indicates freshwater, but higher formation electrical conductivities seen in the upper part of the mud indicate downward saltwater transport from the overlying Holocene marine mud (Fig. 5, borehole Q109). This asymmetrical log pattern can be interpreted as the effect of downward diffusion of marine porewater constituents into the Pleistocene clay, or an advective groundwater flow during Holocene transgression with a sea-level stand in a submerged delta plain. The original marine pore-water in the Pleistocene marine mud had been leached out prior to the deposition of the Holocene muds, most likely due to recharge of fresh-water during low eustatic sea-level stands during glaciations. Numerical modelling explains this diffusion-controlled transport of the original marine porewater out of the mud, as the leaching process is faster where the transport distance is shorter in thin clay layers^8^, as shown with these observations. The widespread presence of marine porewater in the Holocene mud was further documented using transient electromagnetic soundings (TEM) soundings (Supplementary Information, Fig. S1).

**Fig. 6:**
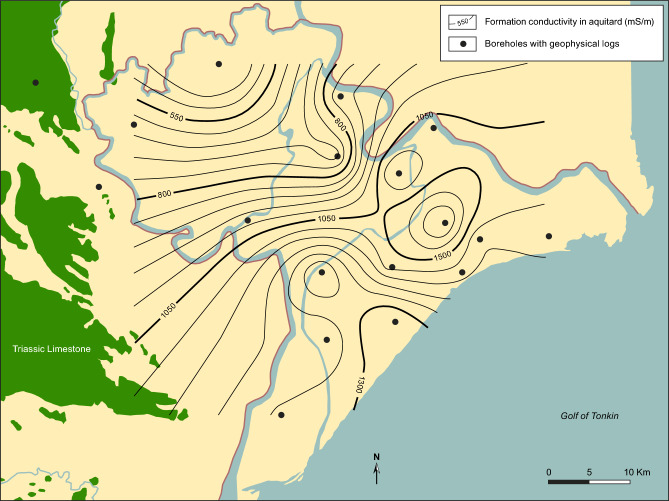
Formation conductivity (mS/m) in the Holocene aquitard interpreted from induction logs. A formation electrical conductivity of 550 mS/m and 1500 mS/m corresponds to pore water chloride concentrations of 5000 mg/L and 16,000 mg/L, respectively. For comparison is the chloride concentration in oceanic water 18,900 mg/L. Map background produced in MapInfo Pro 15.0 using data from National Center for Water Resources Planning and Investigation. Contours produced by Surfer v. 24.

Measured maximum formation conductivities in the marine Holocene muds, as derived from geophysical logging of boreholes, testify to a gradual decrease in salinity inland (Fig. 6). This reflects a decreasing thickness of the mud inland and an increasing sediment age and thus longer time to transport the marine pore-water out of the clay by diffusion^8^. Diffusion profiles are seen in all investigated boreholes, except the most landward borehole Q221. The fact that diffusive profiles, with pristine pore-water present in the mud, are recorded in virtually the entire Nam Dinh province, supported by Darcian calculations and numerical modelling^8^, shows that advective flow, and thus recharge, has not occurred through the delta plain sediment into the Pleistocene during the Holocene. The overall distribution of saline and fresh groundwater in the deep aquifer in this part of the Red River delta is controlled by a diffusive leakage of saline porewater from Holocene marine mud deposited in an incised valley underlying the present Red River^8^. Data from this study confirms this saline and fresh groundwater distribution in the deep aquifer with high groundwater TDS close to the river (Fig. 1). Groundwater ^14^C ages in the deep aquifer are between 5900 and 14,500 years (Supplementary Information Table S1), the ^14^C age of the groundwater is younger closer to the recharge area in the outcropping limestones west and southwest of the Nam Dinh province (Fig. 2). All water samples from the deep aquifer have tritium concentrations below the detection limit (Supplementary Information Table S1). Groundwater samples from the shallow boreholes yield young groundwater with ^3^H values comparable to concentrations in rainwater (Supplementary Information Table S2). Carbon-14 was thus only measured in a few boreholes to verify young groundwater, and these confirmed modern ages. Variations in concentrations of Cl, Na, Br (Supplementary Information Table S2) and δ^18^O in the shallow groundwater show mixing of fresh groundwater and seawater from the shallow channels as the controlling process.

## Broader impacts

In the last decade, increasing focus on the hydrogeological conditions in Quaternary delta systems has been triggered by threats of saltwater pollution to groundwater caused by overexploitation of water resources and climatic changes^2,3,32^. In these deltaic multi-aquifer systems, with present-day low-hydraulic gradient settings, groundwater flow is characterized by slow, and predominantly horizontal flow in aquifers. Hydraulic heads in both shallow aquifers, hydraulically connected to surface water bodies, and in deep aquifers that continue offshore^33,34^, are close to sea-level; hydraulic gradients across aquitards are thus insignificant during the present climate conditions. Given typical hydraulic conductivities in marine aquitard sediments of 10^–10^ m/s^35^, advective transport through the muds is insignificant. The dominant transport mechanism in these multi-aquifer aquitards is by a slow diffusion of the marine pore-water out of the muds, and horizontal transport of the solutes to the sea through the aquifer in a low-gradient environment^8^.

Flushing of pore water in Pleistocene marine aquitards occurred when large hydraulic gradients were established during ~ 100 kyr glacially-generated Milankovitch cycles, with sea-level falls of up to 130–140 m^36,37^. In the RRDP, the presence of freshwater in the 130 kyr-old, deeply buried marine Pleistocene low-permeable formation implies that it was flushed by freshwater when the coast-line of the RRDP migrated out on the shelf, and deep channels were eroded in the delta plain and shelf sediments by rivers^38^.

Lyell’s principle of uniformitarianism implies that by studying present (hydro)geological systems, we can understand these processes in the past. However, applying this principle in coastal, multi-aquifer systems, which experienced rapid glaciations that generated changes in sea level is, not straightforward. Aquitards are low-permeable formations which can transmit significant quantities of water under ordinary hydraulic gradients, but the hydraulic gradients we observe today have not always prevailed in the past. On human-time scale, the Holocene marine aquitards do not transmit significant quantities of water, which contradicts the accepted wisdom that aquitards always leak. The present day fresh-salt groundwater distribution in the Red River delta plain can only be understood if we consider paleo-groundwater flow, and this seems to apply to many of the Quaternary delta systems worldwide^3^. Our use of groundwater resources in delta systems must be adjusted to this knowledge, and groundwater management must also consider that fresh groundwater resources are often located in paleochannels eroded into Pleistocene sediments under low eustatic sea-level stands during glacial periods^38–41^.

Intensive groundwater pumping from deep aquifers might cause downward movement of saline porewater from relatively high permeability Holocene marine muds, as reported from North China Plain^11^. There is no indication of a generated leakage induced from the intensive pumping in this part of the Red River delta, most likely due to the measured very low hydraulic conductivities in the marine mud^8^. If a leakage should be generated in this hydrogeological setting, our study shows that the geological age of the confining marine mud is essential. Holocene marine mud will leak saline porewater, whereas flushed Pleistocene marine mud will leak fresh or brackish water.

## Methods

Data from monitoring boreholes in the Vietnamese National Groundwater Monitoring network and new exploratory boreholes were used to establish the geological model. Robertson Geologging Ltd. equipment was used for geophysical logging of boreholes, and sediment natural gamma radiation and formation electrical conductivity were measured. Formation electrical conductivities were recorded from inside borehole PVC casings using a focused induction probe, which has a formation penetration depth of approximately 5 m. Mapping of the spatial distribution of saltwater in the Red River Delta Plain was performed using the transient electromagnetic method^40^. We used a PROTEM 47 (Geonics Ltd.) with a 40 m by 40 m transmitter loop in a central loop configuration. Current levels of the transmitter were between 0.3 A and 3.0 A producing a maximum magnetic moment of 4800 Am^2^, and the turn-off time for the current was 2.5 µs. This relatively short turn-off time, in combination with early time windows, allows for a proper description of the resistivity properties of the uppermost parts of the subsurface. The decay of the secondary magnetic field recorded by the receiver coil was sampled over three segments to handle the high dynamic range of the received signal. For each segment, measurements were made in 20-time windows (gates). Initial noise tests showed that the signal/noise level was very high in the study area. The initial transient electromagnetic (TEM) data processing, i.e. editing of data and assignment of data uncertainties, was done utilizing the HGG-SiTEM/Semdi software^42,43^. Subsequently, the TEM data were inverted to obtain 1-D resistivity models of the subsurface using a laterally constrained inversion (LCI) scheme^44^. The LCI approach links 1-D resistivity models using a soft constraint on the layer resistivity and layer boundaries. The constraints can be seen as the initial value for the expected geological variations between soundings.

Water sampling was done using a Grundfos MP-1 pump after flushing of boreholes. Immediately after sampling, the pore-water samples were filtered through Sartorius Minisart cellulose acetate filters (0.45 µm). Water samples for determination of stable isotopic composition were not filtered. Water samples were analysed as follows. Water samples for analysis of Na, K, Ca and Mg content were preserved by addition of 2% of a 7 M HNO_3_ solution and refrigerated until analysed by flame absorption spectrophotometry on a Shimadzu AAS 6800 instrument. Samples taken for analysis of Cl, NO_3_ and SO_4_ content were collected in polypropylene vials and frozen immediately after sampling. The anions were analysed by ion chromatography using a Shimadzu LC20AD/HIC-20ASuper. Due to high salinities in the samples, up to 250-fold dilution was required. An 18 MΩ·cm deionized water was used in the dilutions. Pore-water stable isotope ratios of oxygen (^18^O/^16^O) and hydrogen (D) (^2^H/^1^H) were measured relative to the VSMOW standard using a Picarro Cavity Ring-Down Spectrometer (CRDS)^45^ equipped with an autosampler and a vaporiser. The results are expressed in ‰ units using the δ-notation with standard deviations not larger than ± 0.2‰ (δ^18^O) and ± 0.5‰ (for δD), as calculated from four replicate injections into the vaporizer. Sampling for ^3^H was done in HDPE bottles and analysis was done by liquid scintillation counting at the Institute for Nuclear Science and Technology in Hanoi (VNEST). Samples for ^14^C and ^13^C isotopes of dissolved inorganic carbon in water were collected in glass bottles and BaCO_3_ was added to the samples. Analysis was done by liquid scintillation counting at VNEST, using standard procedures, see Supplementary Information.

## Supplementary Information

Supplementary Information.

## Data Availability

Data is provided within the manuscript or supplementary information files.
