# Supplementary material for: Paleo-climatic control on recharge and fresh-salt groundwater distribution in the Red River delta plain, Vietnam
Source: Sci Rep. 2024 Sep 11;14:21280. doi: 10.1038/s41598-024-71899-x (PMC11391079; doi:10.1038/s41598-024-71899-x)
Supplement: Supplementary file 1 — Supplementary Information. [file 41598_2024_71899_MOESM1_ESM.docx]

Supplementary Information

Paleo-climatic control on recharge and fresh-salt groundwater distribution in the Red River delta plain, Vietnam

Flemming Larsen, Hoan Van Hoang, Long Vu Tran, and Nhan Quy Pham

***Results from the transient electromagnetic (TEM) soundings:***

Five layers were used in the laterally constrained inversion (LCI) models, and data fits well within the noise levels for all profiles. One of the four profiles is shown in Fig. S1, and the location of the profile is given in Fig. 1.

E F

VA2 VA1 Q226


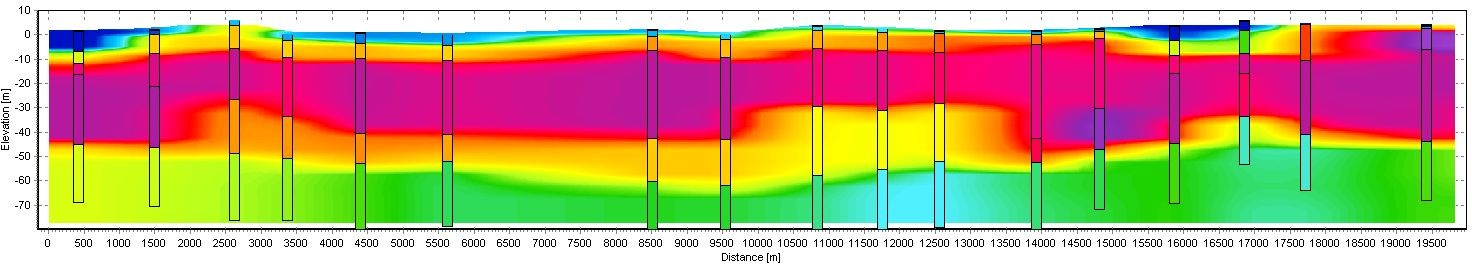

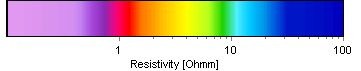


*Fig. S1. TEM survey profile, for location see Fig. 1.*

The illustrated profile is located perpendicular to the coastline, oriented N–S (Fig. 1). The length of the geological cross-section in this profile is 20 km. Near-surface lenses with high resistivities up to 30–40 Ωm are seen in the profile, representing the upper, sandy aquifer. Below this layer and down to 50–60 m, very low resistivities as low as 1-3 Ωm were recorded. This layer represents aquitard clay containing marine porewater. Higher resistivities are present below this layer (10 to 40 Ωm), representing Pleistocene and Neogene aquifers with fresh and brackish groundwater.

**Water stable isotope composition in the Red River**

The variation of δ^18^O in the Red River water samples during 2006 show values between –11 ‰ and –7 ‰. During the dry season from December to July that year, δ^18^O values in the river water were between –9 ‰ and –7 ‰. After the monsoon rain in July, the Red River water becomes more δ^18^O negative in the range –11 ‰ to –9 ‰.


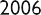

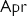

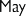

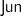

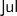

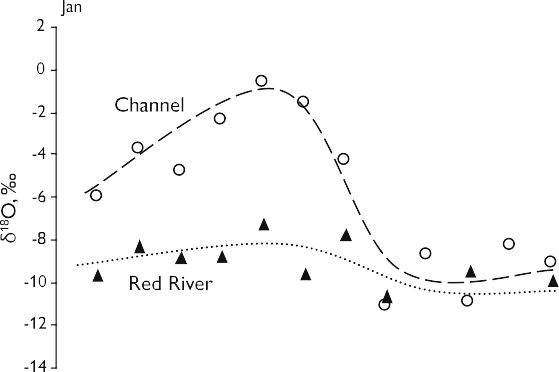

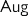

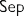


Fig. S2. Reproduced from Larsen et al. 2008: Controlling geological and hydrogeological processes in an arsenic contaminated aquifer on the Red River flood plain, Vietnam.

**Methods used for ^14^C dating:**

Measurements for ^14^C dating were done at Institute for Nuclear Science and Technology in Hanoi (INST).

100 L of groundwater was collected to satisfy the sensitivity of the analysis. *LOD* or *Minimum Detectable Activity* (MDA) in Liquid Scintillation Counting is estimated based on the formula:

$MDA= \frac{4.66xB^{1/2}}{\varepsilon xVxtxX}$

Where *B* is the count number of total background during total counting time t (CPS: counts per second); ε is counting efficiency (CPS/DPS); *V* is the volume of sample to be measured (L); *X* is the probability of radioactive decay.

For ^14^C activity counting on the TriCarb 3770:

- the ε is 0.95; V is 10 ml (+10 ml cocktail accommodated in vials of 20 ml capacity);

- X = 1 (100 % ^14^C decay to ^14^N);

- The measurement was repeated for 10 cycles each prolongs 60 min that the t = 10.60 = 600 min = 36,000 sec.

- For this time, total number of background counting was within 650 counts.

MDA = 4.66x650^1/2^/0.95x10.10^-3^x36,000 = 4.66x25.5/0.95x360 = 0.35 Bq L^-1^

The uncertainty in the ^14^C activity measurement is estimated based on the propagation law of error as follows:

$U= {(\sigma}_{V}^{2}+ \sigma_{CR}^{2}+\sigma_{AM}^{2})$^1/2^

where $\sigma_{V}^{2}$ is error in the volume of water collected for the analysis; ${\sigma2}_{CR}$ is error related to chemical recovery of bicarbonate and carbonate species from the precipitation with barium ions; and ${\sigma2}_{AM}$ is error in radioactivity measurement.

The parameter $\sigma_{V}^{2 t}$is supposed to be 1 % (0.01 or 1 liter per 100 L); $\sigma_{CR}$: very low because precipitation of carbonate barium always achieved quantitatively, and the value can be ignored; $\sigma_{AM}$: 0.1 (10 % precision) if the ^14^C activity is within a range of 50 to 100 pMC (percent of Modern Carbon), and 0.20 if A(^14^C) is lower than 50 pMC.

In case A(^14^C)>50 pMC, the U = (0.01^2^ + 0.1^2^)^1/2^= 0.1 (10 % of mean value)

In case A(^14^C)<50 pMC, the U = (0.01^2^ +0.2^2^)^1/2^ = 0.2 (20 % of mean value)

Carbon-14 activity is usually reported in pMC; this means that in groundwater dating using the ^14^C-dating technique, the ^14^C activity in a sample is measured relatively to the International Standard.

${}^{14}{a= \frac{{{}^{14}A}_{sample}}{{}^{14}{A_{std}}}}$ x 100 pMC

The standard used in this measurement is *oxalic acid* (batch 2) made from French beet molasses planted in 1977 with an absolute ^14^C activity of 0.2147 Bq/g C and the value of δ^13^C = –25 ‰ and called modern carbon.

The “absolute” age (^14^t) of groundwater is estimated based on the formula:

${}^{14}{t= \frac{{}^{14}{T_{1/2}}}{ln2} x \frac{{}^{14}{a_{input}^{0}}}{{}^{14}{a_{sample}}}}$ , y (BP: Before Present, before 1977)

^14^T_1/2_: half-life of ^14^C, 5700 years

${}^{14}{a_{input}^{0}}$ is the initial ^14^C-content in the DIC before entering the saturated zone (pMC), and ^14^a_sample_ is the ^14^C-content (pMC) in the DIC of the sample to be measured.

${}^{14}{a_{input}^{0}}$ is estimated based on the model developed by Gonfiantini for confined aquifer as follows:

$${}^{14}{a_{input}^{0}}= \frac{\delta{}^{13}{C_{DIC}- {}^{13}{C_{cc}}}}{\delta{}^{13}{C_{{CO}_{2}}}, org- {}^{13}{C_{cc}+ \varepsilon_{{CO}_{2}/DIC}}}$$

where *δ^13^C_DIC_, δ^13^C_cc_, δ^13^C_CO2_*, org are the carbon-13 compositions, respectively, in the DIC of a groundwater sample, in calcareous materials in soil/sediment and in the biogenic dioxide originated from the decomposition of organic matters; ε_CO2/DIC_ is the fractionation coefficient for ^13^C in the isotopic exchange reaction between the biogenic carbon dioxide and DIC which is temperature-dependent and determined by the formula:

$\varepsilon_{{CO}_{2}/DIC}=(-\frac{9484}{T}$ + 23.89), ‰

where *T* is the temperature of a groundwater sample in Kelvin. To convert ^14^C content in pMC to “absolute” age, one has to have the data of δ^13^C in DIC, δ^13^C in calcite mineral present in the formation of the aquifer; δ^13^C_CO2, org_ that can be assigned to be –23 ‰ that characterises carbon dioxide generated from mineralisation of remnants of C3 plants in tropical areas followed by its diffusion, the value of ε_CO2/DIC_ varies between 7–8 ‰. Thus,

${}^{14}t=8268xln\frac{{}^{14}{a_{input}^{0}}}{{}^{14}{a_{sample}}}$, y BP

**Porewater composition in the shallow Holocene and deep Pleistocene aquifer**

The porewater chemical compositions in the deep, Pleistocene aquifer and the shallow Holocene aquifer are shown in Tables S1 and S2, respectively.

| *Borehole* | *Depth* |  | *Field data* | | | | | *Laboratory data* | | | | | | | | | | | | | |
| --- | --- | --- | --- | --- | --- | --- | --- | --- | --- | --- | --- | --- | --- | --- | --- | --- | --- | --- | --- | --- | --- |
| *No* | *m* | *Aquifer* | *EC* | *T,* | *pH* | *Fe(II)* | *HCO_3_^-^* | *Na^+^* | *K^+^* | *Ca^2+^* | *Mg^2+^* | *NH4* | *Cl^-^* | *NO_3_^-^* | *SO_4_^2-^* | *δ^18^O* | *δ^2^H* | *^3^H* | *^13^C_TDIC,_ ‰* | *^14^C_TDIC,_* | *^14^C age* |
|  |  |  | *µS/cm* | *^o^C* |  | *mg/L* | | | | | | | | | | *‰* | *‰* | *TU* | *vs. VPDB* | *pMC* | *a BP* |
| *Q92* | *43.0* | *qp* | *825* | *27.4* | *7.6* | *2.6* | *253.4* | *30.5* | *5.1* | *48.7* | *56.8* | *0.3* | *185.0* | *0.1* | *0.5* | *-5.9* | *-38.0* | *<LOQ* | *-12.9* | *74.6±2.1* | *1100 ± 50* |
| *Q92a* | *100.0* | *t* | *1032* | *26.5* | *8.1* | *0.4* | *83.2* | *87.9* | *4.7* | *51.3* | *55.5* | *0.2* | *276.0* | *0.1* | *0.2* | *-7.1* | *-42.6* | *<LOQ* | *-8.9* | *54.1±2.5* | *850 ± 40* |
| *Q108b* | *80.0* | *qp* | *1005* | *27.6* | *7.0* | *2.5* | *172.5* | *93.2* | *20.6* | *12.7* | *27.3* | *1.4* | *215.0* | *0.2* | *1.1* | *-6.9* | *-47.2* | *<LOQ* | *-11.4* | *51.1±1.4* | *3300 ± 90* |
| *Q109a* | *135.8* | *qp* | *2136* | *24.2* | *7.3* | *1.7* | *572.4* | *386.6* | *9.7* | *44.9* | *61.1* | *6.3* | *453.0* | *0.1* | *0.5* | *-7.2* | *-50.2* | *<LOQ* | *-11.6* | *19.2±2.7* | *11300 ± 1600* |
| *Q109b* | *170.6* | *n* | *1547* | *27.6* | *7.5* | *0.2* | *236.8* | *227.3* | *15.6* | *13.5* | *28.5* | *2.6* | *347.0* | *0.05* | *1.4* | *-6.7* | *-46.3* | *<LOQ* | *-11.6* | *30.8±2.0* | *7400 ± 500* |
| *Q110a* | *93.6* | *qp* | *957* | *27.7* | *7.3* | *0.7* | *275.9* | *115.6* | *3.2* | *41.3* | *33.5* | *0.4* | *164.0* | *0.1* | *2.7* | *-6.6* | *-47.3* | *<LOQ* | *-11.4* | *36.2±1.8* | *6000 ± 300* |
| *Q220T* | *100.0* | *t* | *763* | *27.4* | *7.5* | *0.7* | *248.8* | *157.3* | *5.7* | *1.5* | *1.7* | *2.6* | *30.6* | *0.1* | *57.9* | *-6.7* | *-42.4* | *<LOQ* | *-12.0* | *47.8±1.7* | *3700 ± 130* |
| *Q221a* | *70.0* | *qp* | *2836* | *26.9* | *6.9* | *6.9* | *557.9* | *293.6* | *9.9* | *102.7* | *70.4* | *34.6* | *765.2* | *0.2* | *0.6* | *-7.6* | *-59.5* | *<LOQ* | *-14.1* | *41.6±1.7* | *5900 ± 250* |
| *Q221n* | *127.0* | *n* | *5428* | *27.4* | *7.0* | *1.5* | *403.6* | *795.8* | *15.4* | *112.2* | *106.2* | *7.4* | *1572.0* | *0.1* | *12.4* | *-7.6* | *-59.0* | *<LOQ* | *-12.9* | *21.6±2.2* | *11300 ± 1150* |
| *Q222b* | *115.0* | *qp* | *6297* | *26.8* | *6.7* | *24.7* | *137.7* | *1146.3* | *13.7* | *106.3* | *125.5* | *45.6* | *2247.0* | *0.4* | *0.7* | *-6.6* | *-42.7* | *<LOQ* | *-13.0* | *28.3±2.5* | *9100 ± 800* |
| *Q223n* | *138.0* | *n* | *2548* | *29.1* | *6.5* | *11.6* | *145.7* | *364.7* | *8.8* | *99.8* | *87.4* | *6.8* | *8436.0* | *0.1* | *1.1* | *-8.6* | *-60.5* | *<LOQ* | *-13.1* | *14.7±2.6* | *14500 ± 2500* |
| *Q224a* | *100.0* | *qp* | *2936* | *27.6* | *6.9* | *13.3* | *189.8* | *254.8* | *13.5* | *117.2* | *92.5* | *4.9* | *826.0* | *0.2* | *0.8* | *-7.4* | *-53.6* | *<LOQ* | *-13.1* | *27.0±2.2* | *9500 ± 750* |
| *Q225a* | *110.0* | *qp* | *6132* | *27.7* | *7.2* | *0.7* | *535.7* | *983.7* | *29.8* | *86.7* | *106.3* | *15.7* | *1768.0* | *0.1* | *1.1* | *-7.3* | *-50.0* | *<LOQ* | *-11.4* | *23.3±3.2* | *9700 ± 1300* |
| *Q226a* | *105.0* | *qp* | *3563* | *28.4* | *7.3* | *4.8* | *489.2* | *604.9* | *22.5* | *51.2* | *45.7* | *21.2* | *1023.0* | *0.1* | *0.3* | *-7.0* | *-43.9* | *<LOQ* | *-11.4* | *21.8±2.2* | *10200 ± 1000* |
| *Q226n* | *151.5* | *n* | *2839* | *29.6* | *7.1* | *3.9* | *373.7* | *297.5* | *10.6* | *123.5* | *80.9* | *3.7* | *778.0* | *0.1* | *0.2* | *-7.7* | *-52.4* | *<LOQ* | *-13.2* | *16.6±2.6* | *13500 ± 2100* |
| *Q227a* | *155.5* | *qp* | *397* | *28.7* | *7.5* | *0.8* | *254.6* | *92.6* | *4.5* | *13.1* | *25.4* | *0.5* | *17.3* | *0.2* | *7.5* | *-7.7* | *-51.7* | *<LOQ* | *-11.5* | *15.8±2.6* | *12900 ± 2000* |
| *Q228a* | *120.0* | *qp* | *405* | *28.5* | *7.8* | *0,.5* | *263.6* | *132.4* | *3.7* | *1.4* | *3.7* | *0.7* | *15.9* | *0.4* | *0.5* | *-7.8* | *-65.7* | *<LOQ* | *-11.5* | *18.9±3.5* | *11400 ± 2100* |
| *Q229a* | *85.0* | *qp* | *479* | *27.4* | *7.8* | *0.8* | *277.6* | *111.8* | *5.8* | *11.6* | *15.8* | *0.7* | *25.8* | *0.2* | *0.6* | *-6.3* | *-40.5* | *<LOQ* | *-11.4* | *52.8±1.6* | *2900 ± 87* |
| *Q229n* | *150.0* | *n* | *439* | *28.7* | *7.4* | *0.2* | *266.8* | *74.7* | *5.5* | *4.7* | *14.6* | *0.3* | *24.8* | *0.2* | *0.6* | *-7.1* | *-53.4* | *<LOQ* | *-12.0* | *16.7±2.1* | *12400 ± 1600* |
| *ND-01* | *132.0* | *qp* | *3400* | *29.4* | *6.9* | *9.0* | *295.9* | *378.0* | *87.4* | *87.4* | *70.0* | *8.4* | *780.0* | *1.3* | *0.0* | *Nd* | *nd* | *nd* | *Nd* | *nd* |  |
| *ND-02* | *139.0* | *qp* | *14050* | *28.2* | *7.0* | *15.4* | *500.2* | *2151.0* | *207,8* | *207,8* | *205.6* | *65.8* | *3988.0* | *1.7* | *0.0* | *Nd* | *nd* | *nd* | *Nd* | *nd* |  |

**Supplementary Information Table S1.** *qp* = Pleistocene aquifer, *n* = Neogene aquifer, *nd =* no data, *LOQ* = Limit of Quantification.

| \| *Borehole* \| *Depth* \| *Field data* \| \| \| \| \| \| \| *Laboratory data* \| \| \| \| \| \| \| \| \| \| \| \| \| \| \| \| \| --- \| --- \| --- \| --- \| --- \| --- \| --- \| --- \| --- \| --- \| --- \| --- \| --- \| --- \| --- \| --- \| --- \| --- \| --- \| --- \| --- \| --- \| --- \| --- \| --- \| \| *No* \| *m* \| *Ec* \| *T* \| *pH* \| *O2* \| *Fe(II)* \| *PO4* \| *HCO3-* \| *Na* \| *K* \| *Ca* \| *Mg* \| *Cl* \| *SO4* \| *Br* \| *NO3* \| *F* \| *δO^18^* \| *δD* \| *^3^H* \| *δ^13^C_TDIC,_ ‰* \| *^14^C_TDIC,_* \| *^14^C-age* \| \|  \|  \| *mS/cm* \| *°C* \|  \| *mg/L* \| \| \| \| \| \| \| \| \| \| \| \| \| *‰* \| *‰* \| *TU* \| *vs. VPDB* \| *pMC* \| *a BP* \| \| *OB01 D* \| *7.6* \| *22.5* \| *25.2* \| *7.,5* \| *0.2* \| *18.8* \| *2.6* \| *777.8* \| *3,864.6* \| *108.9* \| *54.8* \| *300.5* \| *7,113* \| *0.6* \| *24.2* \| *0.0* \| *0.4* \| *-4.5* \| *-29.4* \| *2.73±0.32* \| *nd* \| *nd* \| *nd* \| \| *OB02 D* \| *8.5* \| *32.0* \| *25.7* \| *7.4* \| *0.2* \| *38.5* \| *0.6* \| *427.0* \| *5,503.9* \| *99.7* \| *238.7* \| *482.2* \| *5,172* \| *0.6* \| *36.2* \| *0.0* \| *0.2* \| *-4.5* \| *-28.8* \| *2.06±0.37* \| *nd* \| *nd* \| *nd* \| \| *OB03 D* \| *59.5* \| *10.2* \| *26.4* \| *7.1* \| *0.2* \| *20.6* \| *1.8* \| *588.7* \| *1,438.1* \| *28.6* \| *130.4* \| *128.7* \| *3,045* \| *0.2* \| *9.8* \| *0.0* \| *0.1* \| *-6.7* \| *-45.8* \| *2.63±0.36* \| *nd* \| *nd* \| *nd* \| \| *OB04 D* \| *8.3* \| *36.3* \| *24.7* \| *8.0* \| *0.2* \| *5.3* \| *2.1* \| *585.6* \| *6,097.0* \| *199.0* \| *45.3* \| *511.6* \| *8,411* \| *0.4* \| *49.6* \| *0.0* \| *0.7* \| *-3.4* \| *-21.6* \| *3.10±0.33* \| *nd* \| *nd* \| *nd* \| \| *OB06 D* \| *6.7* \| *9.4* \| *25.0* \| *7.9* \| *0.3* \| *1.9* \| *3.0* \| *930.3* \| *1,692.8* \| *74.6* \| *32.9* \| *148.6* \| *2,643* \| *0.3* \| *8.9* \| *0.0* \| *0.3* \| *-6.8* \| *-44.4* \| *2.60±0.33* \| *nd* \| *nd* \| *nd* \| \| *OB07 D* \| *7.3* \| *1.5* \| *25.4* \| *7.4* \| *0.3* \| *12.8* \| *1.4* \| *844.9* \| *165.3* \| *23.7* \| *68.9* \| *55.3* \| *110* \| *11.9* \| *0.5* \| *0.0* \| *0.2* \| *-5.9* \| *-38.9* \| *1.89±0.46* \| *nd* \| *nd* \| *nd* \| \| *OB08 D* \| *8.1* \| *2.6* \| *25.2* \| *7.2* \| *0.3* \| *15.5* \| *2.0* \| *841.8* \| *364.9* \| *20.0* \| *52.1* \| *62.0* \| *537* \| *0.2* \| *2.1* \| *0.0* \| *0.1* \| *-6.1* \| *-41.9* \| *2.55±0.37* \| *nd* \| *Nd* \| *nd* \| \| *OB09 D* \| *6.1* \| *0.2* \| *26.1* \| *7.3* \| *0.1* \| *3.4* \| *0.2* \| *143.4* \| *3.2* \| *1.5* \| *26.8* \| *5.4* \| *3.0* \| *8.2* \| *0.0* \| *0.0* \| *0.1* \| *-5.6* \| *-31.5* \| *2.83±0.26* \| *-17.25* \| *101.2* \| *modern* \| \| *OB10 D* \| *8.0* \| *21.0* \| *25.8* \| *7.6* \| *0.1* \| *11.6* \| *4.4* \| *1,525.0* \| *3,344.8* \| *147.3* \| *73.9* \| *392.5* \| *6,101* \| *0.6* \| *20.1* \| *0.0* \| *0.5* \| *-5.2* \| *-35.5* \| *2.31±0.41* \| *nd* \| *nd* \| *nd* \| \| *OB11 D* \| *7.8* \| *11.4* \| *25.5* \| *7.8* \| *0.1* \| *1.7* \| *5.4* \| *1,433.5* \| *1,951.7* \| *90.2* \| *43.5* \| *188.6* \| *3,047* \| *0.4* \| *10.5* \| *0.0* \| *0.3* \| *-4.0* \| *-29.4* \| *2.03±0.36* \| *-17.32* \| *103.5* \| *modern* \| \| *OB12 D* \| *7.6* \| *4.3* \| *25.8* \| *7.5* \| *0.1* \| *13.2* \| *1.4* \| *817.4* \| *680.9* \| *35.8* \| *77.2* \| *95.8* \| *1,118* \| *0.2* \| *3.9* \| *0.0* \| *0.1* \| *-6.0* \| *-41.5* \| *1.93±0.43* \| *nd* \| *Nd* \| *nd* \| \| *OB13 D* \| *6.7* \| *38.7* \| *25.4* \| *7.6* \| *0.1* \| *8.7* \| *3.8* \| *2,000.8* \| *6,424.6* \| *220.1* \| *129.3* \| *679.6* \| *11,236* \| *3.8* \| *65.8* \| *0.0* \| *0.5* \| *-3.3* \| *-21.9* \| *2.02±0.37* \| *nd* \| *nd* \| *nd* \| \| *OB14 D* \| *8.4* \| *21.7* \| *24.3* \| *7.5* \| *0.5* \| *5.2* \| *5.6* \| *1,403.0* \| *3,392.2* \| *141.7* \| *47.5* \| *305.0* \| *6,482* \| *0.6* \| *21.3* \| *0.0* \| *0.5* \| *-4.1* \| *-27.5* \| *2.04±0.32* \| *nd* \| *nd* \| *nd* \| \| *OB15 D* \| *8.8* \| *6.9* \| *28.7* \| *7.0* \| *0.4* \| *29.4* \| *1.5* \| *756.4* \| *962.4* \| *29.0* \| *121.5* \| *97.2* \| *736* \| *0.2* \| *2.9* \| *0.0* \| *0.0* \| *-5.1* \| *-34.5* \| *2.52±0.33* \| *nd* \| *nd* \| *nd* \| \| *OB16 D* \| *9.6* \| *1.8* \| *25.0* \| *7.5* \| *0.1* \| *2.5* \| *2.3* \| *771.7* \| *207.4* \| *38.2* \| *44.8* \| *82.8* \| *275* \| *0.1* \| *0.9* \| *0.0* \| *0.4* \| *-3.9* \| *-29.0* \| *nd* \| *nd* \| *nd* \| *nd* \| \| *OB17 D* \| *13.8* \| *21.2* \| *28.1* \| *7.7* \| *0.3* \| *7.3* \| *5.7* \| *1,274.9* \| *3,544.2* \| *149.7* \| *33.8* \| *301.8* \| *6,596* \| *0.2* \| *21.9* \| *0.0* \| *0.4* \| *-4.5* \| *-30.4* \| *nd* \| *nd* \| *nd* \| *nd* \| \| *OB18 D* \| *7.1* \| *2.8* \| *24.7* \| *7.9* \| *0.2* \| *1.7* \| *1.4* \| *634.4* \| *408.3* \| *29.2* \| *46.0* \| *68.9* \| *630* \| *0.4* \| *2.2* \| *0.0* \| *0.2* \| *-4.1* \| *-27.7* \| *nd* \| *nd* \| *nd* \| *nd* \| \| *OB19 D* \| *7.4* \| *0.3* \| *25.7* \| *7.1* \| *0.1* \| *21.8* \| *0.4* \| *158.6* \| *17.9* \| *5.1* \| *14.0* \| *5.7* \| *9.1* \| *0.1* \| *0.0* \| *0.0* \| *0.1* \| *-4.9* \| *-34.0* \| *nd* \| *nd* \| *nd* \| *nd* \| \| *OB20 D* \| *6.5* \| *2.4* \| *25.2* \| *6.9* \| *0.2* \| *36.7* \| *1.5* \| *555.1* \| *154.9* \| *9.9* \| *161.8* \| *51.5* \| *537* \| *0.2* \| *2.3* \| *0.0* \| *0.1* \| *-6.6* \| *-45.3* \| *nd* \| *nd* \| *nd* \| *nd* \| \| *OB21 D* \| *7.9* \| *1.7* \| *23.8* \| *7.5* \| *0.8* \| *2.8* \| *1.1* \| *603.9* \| *152.3* \| *35.2* \| *51.3* \| *81.6* \| *267* \| *0.4* \| *0.9* \| *0.0* \| *0.5* \| *-3.0* \| *-19.8* \| *nd* \| *nd* \| *nd* \| *nd* \| \| *ND 02a* \| *15.0* \| *330.0* \| *Nd* \| *7.5* \| *nd* \| *8.7* \| *nd* \| *695.4* \| *484.4* \| *15.1* \| *123.8* \| *50.3* \| *793* \| *0.2* \| *3.1* \| *0.0* \| *0.1* \| *-7.2* \| *-48.3* \| *nd* \| *nd* \| *nd* \| *nd* \| |
| --- | --- | --- | --- | --- | --- | --- | --- | --- | --- | --- | --- | --- | --- | --- | --- | --- | --- | --- | --- | --- | --- | --- | --- | --- | --- | --- | --- | --- | --- | --- | --- | --- | --- | --- | --- | --- | --- | --- | --- | --- | --- | --- | --- | --- | --- | --- | --- | --- | --- | --- | --- | --- | --- | --- | --- | --- | --- | --- | --- | --- | --- | --- | --- | --- | --- | --- | --- | --- | --- | --- | --- | --- | --- | --- | --- | --- | --- | --- | --- | --- | --- | --- | --- | --- | --- | --- | --- | --- | --- | --- | --- | --- | --- | --- | --- | --- | --- | --- | --- | --- | --- | --- | --- | --- | --- | --- | --- | --- | --- | --- | --- | --- | --- | --- | --- | --- | --- | --- | --- | --- | --- | --- | --- | --- | --- | --- | --- | --- | --- | --- | --- | --- | --- | --- | --- | --- | --- | --- | --- | --- | --- | --- | --- | --- | --- | --- | --- | --- | --- | --- | --- | --- | --- | --- | --- | --- | --- | --- | --- | --- | --- | --- | --- | --- | --- | --- | --- | --- | --- | --- | --- | --- | --- | --- | --- | --- | --- | --- | --- | --- | --- | --- | --- | --- | --- | --- | --- | --- | --- | --- | --- | --- | --- | --- | --- | --- | --- | --- | --- | --- | --- | --- | --- | --- | --- | --- | --- | --- | --- | --- | --- | --- | --- | --- | --- | --- | --- | --- | --- | --- | --- | --- | --- | --- | --- | --- | --- | --- | --- | --- | --- | --- | --- | --- | --- | --- | --- | --- | --- | --- | --- | --- | --- | --- | --- | --- | --- | --- | --- | --- | --- | --- | --- | --- | --- | --- | --- | --- | --- | --- | --- | --- | --- | --- | --- | --- | --- | --- | --- | --- | --- | --- | --- | --- | --- | --- | --- | --- | --- | --- | --- | --- | --- | --- | --- | --- | --- | --- | --- | --- | --- | --- | --- | --- | --- | --- | --- | --- | --- | --- | --- | --- | --- | --- | --- | --- | --- | --- | --- | --- | --- | --- | --- | --- | --- | --- | --- | --- | --- | --- | --- | --- | --- | --- | --- | --- | --- | --- | --- | --- | --- | --- | --- | --- | --- | --- | --- | --- | --- | --- | --- | --- | --- | --- | --- | --- | --- | --- | --- | --- | --- | --- | --- | --- | --- | --- | --- | --- | --- | --- | --- | --- | --- | --- | --- | --- | --- | --- | --- | --- | --- | --- | --- | --- | --- | --- | --- | --- | --- | --- | --- | --- | --- | --- | --- | --- | --- | --- | --- | --- | --- | --- | --- | --- | --- | --- | --- | --- | --- | --- | --- | --- | --- | --- | --- | --- | --- | --- | --- | --- | --- | --- | --- | --- | --- | --- | --- | --- | --- | --- | --- | --- | --- | --- | --- | --- | --- | --- | --- | --- | --- | --- | --- | --- | --- | --- | --- | --- | --- | --- | --- | --- | --- | --- | --- | --- | --- | --- | --- | --- | --- | --- | --- | --- | --- | --- | --- | --- | --- | --- | --- | --- | --- | --- | --- | --- | --- | --- | --- | --- | --- | --- | --- | --- | --- | --- | --- | --- | --- | --- | --- | --- | --- | --- | --- | --- | --- | --- | --- | --- | --- | --- | --- | --- | --- | --- | --- | --- | --- | --- | --- | --- | --- | --- | --- | --- | --- | --- | --- | --- | --- | --- | --- | --- | --- | --- | --- | --- | --- | --- | --- | --- | --- | --- | --- | --- | --- | --- | --- | --- | --- | --- | --- | --- | --- | --- | --- | --- | --- | --- | --- | --- | --- | --- | --- | --- | --- | --- | --- | --- | --- | --- | --- | --- | --- | --- | --- | --- | --- | --- | --- | --- | --- | --- | --- | --- | --- | --- | --- | --- | --- | --- | --- | --- | --- | --- | --- |

**Supplementary Information Table S2.** Holocene aquifer, *nd* = no data.
